# Redox-state-regulating enzymes have prognostic value in diabetic endometrial cancer patients: impact of statin use?

**DOI:** 10.3389/fonc.2024.1393103

**Published:** 2024-07-08

**Authors:** Elina Ollila, Anne Ahtikoski, Ulla Puistola, Peeter Karihtala, Elina Urpilainen

**Affiliations:** ^1^ Department of Obstetrics and Gynecology, Oulu University Hospital, Wellbeing Services County of North Ostrobothnia, and University of Oulu, Oulu, Finland; ^2^ Research Unit of Clinical Medicine, University of Oulu, Oulu, Finland; ^3^ Medical Research Center, Oulu University Hospital, Wellbeing Services County of North Ostrobothnia, University of Oulu, Oulu, Finland; ^4^ Department of Pathology, Fimlab Laboratories, University of Tampere, Tampere, Finland; ^5^ Department of Oncology, Helsinki University Hospital Comprehensive Cancer Center, University of Helsinki, Helsinki, Finland

**Keywords:** oxidative stress, MnSOD, Nrf2, type 2 diabetes, KEAP1

## Abstract

**Objective:**

The incidence of endometrial cancer is increasing, and oxidative stress has been suggested to play a vital role in its carcinogenesis. Statins have an impact on the cellular redox-state. The aim of this study was to determine the effects of statin use on redox-state regulating enzymes in endometrial cancer in women with type 2 diabetes.

**Materials and methods:**

This retrospective study consisted of 119 women with type 2 diabetes who were diagnosed with endometrial cancer at Oulu University Hospital in Finland between 2007 and 2014. There were 58 statin users and 61 non-users based on medication use at the time of endometrial cancer diagnosis. The expression of redox-state regulating proteins nuclear factor erythroid 2-related factor 2 (Nrf2) and Kelch-like ECH-associated protein 1 (Keap1) in the tumor samples was assessed immunohistochemically, and manganese superoxide dismutase (MnSOD) levels were measured both immunohistochemically and from serum samples.

**Results:**

High MnSOD expression predicted better progression-free survival (PFS) in statin non-users in a univariate analysis (p=0.02). There was no statistical difference in overall survival (OS) or PFS between strong and weak expression of Nrf2 and Keap1. After adjusting for stage and statin use, the results were similar.

**Conclusion:**

Statin non-users with strong MnSOD expression had better PFS compared to statin users which proves that statins have impact on redox-state regulating enzymes. However, these findings are preliminary and require further research.

## 1 Introduction

Endometrial cancer incidence is increasing, most likely due to obesity and type 2 diabetes mellitus (T2DM) (1). Women with type 1 diabetes mellitus and T2DM have twice the risk of developing endometrial cancer as women without diabetes, regardless of weight (2).

Patients with T2DM have a higher risk of death from cardiovascular diseases causes; therefore, statins are widely used among these patients (3). In Finland, 79% of patients with newly diagnosed diabetes use statins for secondary prevention and 40% for primary prevention of cardiovascular diseases (4). It is still debated whether statin use improves the prognosis of endometrial cancer and decreases mortality. One meta-analysis indicated that statin use could increase overall survival (OS) and increase disease-specific survival in patients with endometrial cancer (5).

Cellular redox-state regulating enzymes such as nuclear factor erythroid 2-related factor 2 (Nrf2), Kelch-like ECH-associated protein 1 (Keap1), and manganese superoxide dismutase (MnSOD) have been studied in various cancer types. Nfr2 overexpression is related to endometrial serous carcinomas (6). The Nrf2 pathway is a cellular defensive mechanism against oxidants (7). Nrf2 overexpression is related to worse clinical outcomes in breast cancer (8) and lung cancer (9). A meta-analysis of nine studies of different cancer types also ultimately concluded that Nrf2 overexpression lowers both overall and disease-free survival (10).

Our group has previously reported Keap1 overexpression to be associated with poor prognosis in endometrial cancer (11). Although strong MnSOD expression is related to poorer OS and progression-free survival (PFS) in breast and skin cancer (12, 13), its role in endometrial cancer has yet to be revealed.

This study aimed to explore the role of redox-state regulating proteins according to statin use in patients with endometrial cancer and T2DM.

## 2 Patients and methods

### 2.1 Study cohort

The study population consisted of women with T2DM who were diagnosed with endometrial cancer at Oulu University Hospital in Finland between 2007 and 2014. Women whose statin use information was unknown at the time of diagnosis were excluded from the study. Data collected from hospital records included women’s age at the time of endometrial cancer diagnosis, parity, statin use, antidiabetic medication use, menopause age, presence of fatty liver, and body mass index. Also, cancer-related information, including stage, histology, peritoneal cytology, myometrial invasion, lymphovascular invasion, estrogen receptor status, and the presence of a residual tumor after surgery and adjuvant treatment, was collected from the records.

Women were classified as statin users if they used statin medication at the time of endometrial cancer diagnosis. All endometrial cancer diagnoses were based on histology, and stages were fitted to the International Federation of Gynecology and Obstetrics classification 2009 (14). Stages IA and IB were categorized as early stage, while advanced stages were classified as stages II, III, and IV. The cancers were categorized as type 1 and type 2 cancers according to their histology. Type 1 histology included grades 1 and 2 endometrioid endometrial cancers and mucinous cancers. Type 2 histology included grade 3 endometrioid, serous, clear cell, mixed, and undifferentiated endometrial cancers, as well as carcinosarcomas. Since the cancer diagnostics were conducted prior to the 5^th^ edition of WHO Classification of Tumors (15), only histological classification was used.

Follow-up began at the time of endometrial cancer surgery, except in patients who were ineligible for surgery. In those 14 cases, follow-up began at the time of endometrial cancer diagnosis. Follow-up ended at the time of death or at the closure of the follow-up period (August 7, 2018).

### 2.2 Immunohistochemistry

Keap1, Nrf2, and MnSOD expression were assessed immunohistochemically. The tumor tissue samples were collected during primary surgery. Diagnostic endometrial biopsy was used in cases ineligible for surgery. Tissue sections of 3.5 µm were cut from a representative paraffin block and placed on SuperFrost Plus glass slides (Menzel Gläser, Braunschweig, Germany). At first, the sections were deparaffinized in xylene and rehydrated in a descending series of ethanol concentrations and then rinsed. Antigen retrieval was completed in 10 mM citrate buffer (pH 6.0) in a microwave oven for 4 min, followed by cooling at room temperature. Then, the slides were immersed in 3% hydrogen peroxide in methanol for 15 min to consume the endogenous peroxide.

Immunostaining was performed using rabbit monoclonal anti-Nrf2 (EPI 808Y, Abcam, Cambridge, UK) at 1:300 dilution, goat polyclonal anti-Keap1 (Santa Cruz Biotechnology, Santa Cruz, USA) at 1:100 dilution, and rabbit anti-MnSOD (SigmaAldrich, St. Louis, MO, USA) at 1:1000 dilution. The Dako EnVision kit (Dako, Glostrup, Denmark) was used for the detection of Nrf2, the goat-on-rodent HRP-polymer kit (Biocare GHP516L, Biocare Medical, concord, CA, USA) was used for Keap1, and the Novolink Polymer Detection System (Leica Biosystems Newcastle, Newcastle upon Tyne, UK) was used for MnSOD. Aminoethyl carbazole (Zymed Laboratories, South San Francisco, CA, USA) was used as a chromogen. Meyer’s hematoxylin, immersed in 2% ammonia water, was used for counterstaining, and, finally, the sections were mounted with Immu-Mount (Shandon, Pittsburgh, PA, USA). Negative controls were prepared using the same procedure, except that the primary antibodies were replaced with PBS or serum isotype controls (Zymed Laboratories).

**Figure 1:**
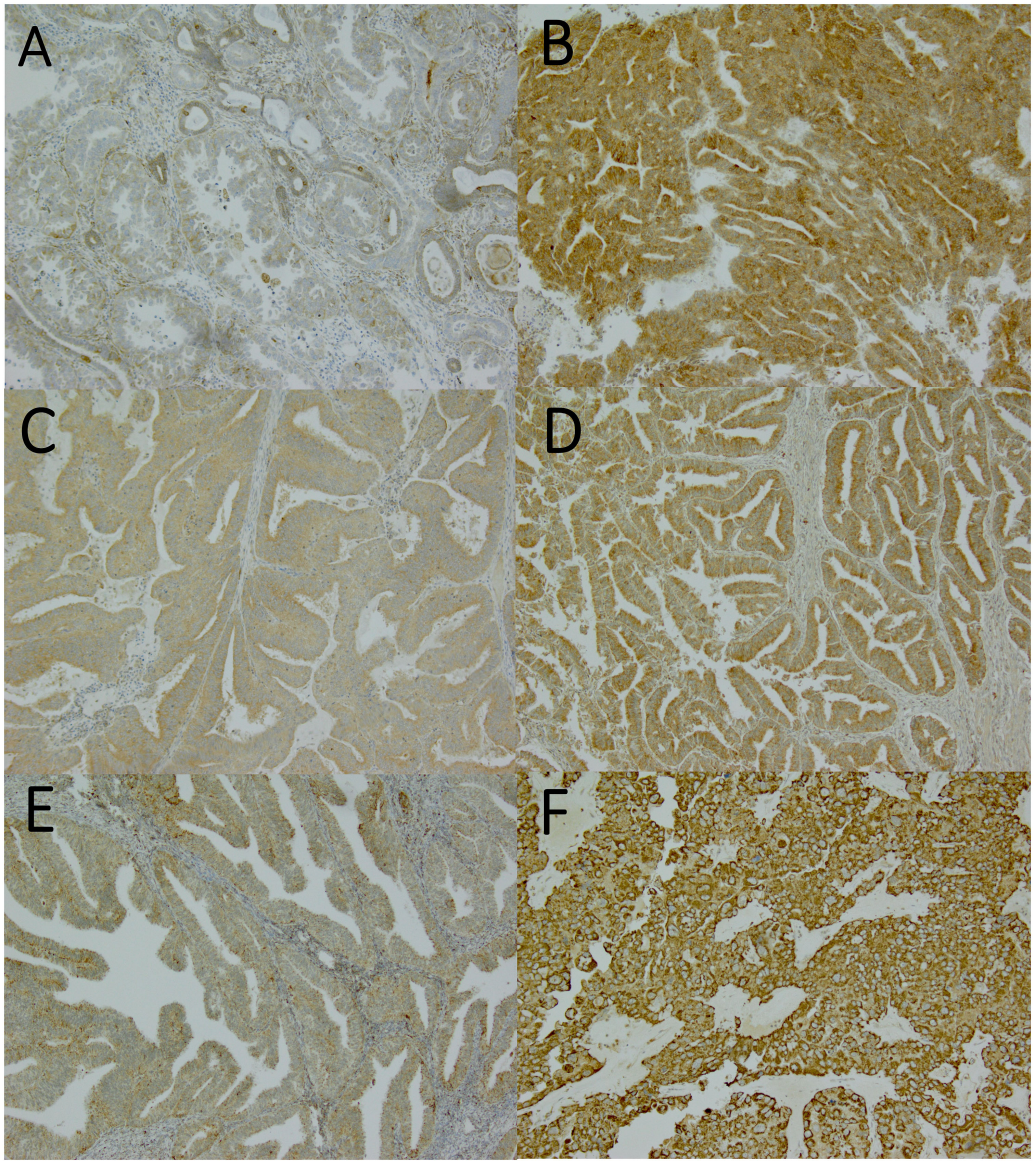
Nuclear factor erythroid 2-related factor 2 (Nrf2), Kelch-like ECH-associated protein 1 (Keap1), and manganese superoxide dismutase (MnSOD) expressions assessed by immunohistochemistry. Magnification x100. **(A)** weak Nrf2 expression (graded as 0), **(B)** strong Nrf2 expression (graded as 2), **(C)** weak Keap1 expression (graded as 1), **(D)** strong Keap1 expression (graded as 2), **(E)** weak MnSOD expression (graded as 1), **(F)** strong MnSOD expression (graded as 3).

Immunoreactivity in the samples was assessed by the staining intensity in the cytoplasm of the tumor cells and the proportion of positively stained tumor cells (**Figure 1**). The immunoreactivity was evaluated by two independent investigators (EU and AA) blinded to the clinical data. When the evaluation of immunoreactivity was different between investigators, the sample was re-evaluated, and consensus was reached.

The staining reactions were categorized into four groups: 0, no staining or only a few positive cells; 1, diffuse weak intensity or moderate staining intensity in maximum of 50% tumor cells; 2, moderate staining intensity >50% with strong intensity <90% of tumor cells; and 3, strong staining intensity at least in 90% of tumor cells). The results were further categorized in two groups: weak (0–1) and strong (2–3) expression (**Figure 1**).

### 2.3 Serum samples

Serum samples were collected during the first hospital visit. The samples were stored at –70°C until analyzed. The design of the serum MnSOD assay was based on a sandwich enzyme-linked immunosorbent assay by using the commercial LF-EK0104 kit (Abfrontier, Seoul, South-Korea) and following the manufacturer’s indicated protocol. The median level of serum MnSOD (1108.6 pg/mL) was used to categorize the results into two groups: low and high.

### 2.4 Statistical methods

Statistical analysis was performed using IBM SPSS Statistics software, version 28.0.1.0, and Kaplan–Meier curves were drawn using GraphPad Prism software, version 8.0.2 (GraphPad Software, San Diego, CA, USA). For patient characteristics, we compared weak and high protein expressions. Continuous variables in the patient characteristics were analyzed using a t-test or a Mann–Whitney test, according to data distribution. Categorical variables in the patient and tumor characteristics were analyzed by Pearson’s chi-square test. The Cox proportional hazards model was used to obtain hazard ratios and 95% confidence intervals in order to investigate the associations between PFS or OS and the redox-state regulating proteins, statin use, and endometrial cancer stage. The assumptions of the Cox regression model were checked graphically by drawing Kaplan–Meier curves. PFS was calculated from the time of the surgery to the date of radiological progression. OS was calculated from the time of the surgery or cancer diagnosis to the time of death from any cause. In all analyses, the statistically significant limit of p was < 0.05.

## 3 Results

**Figure 2:**
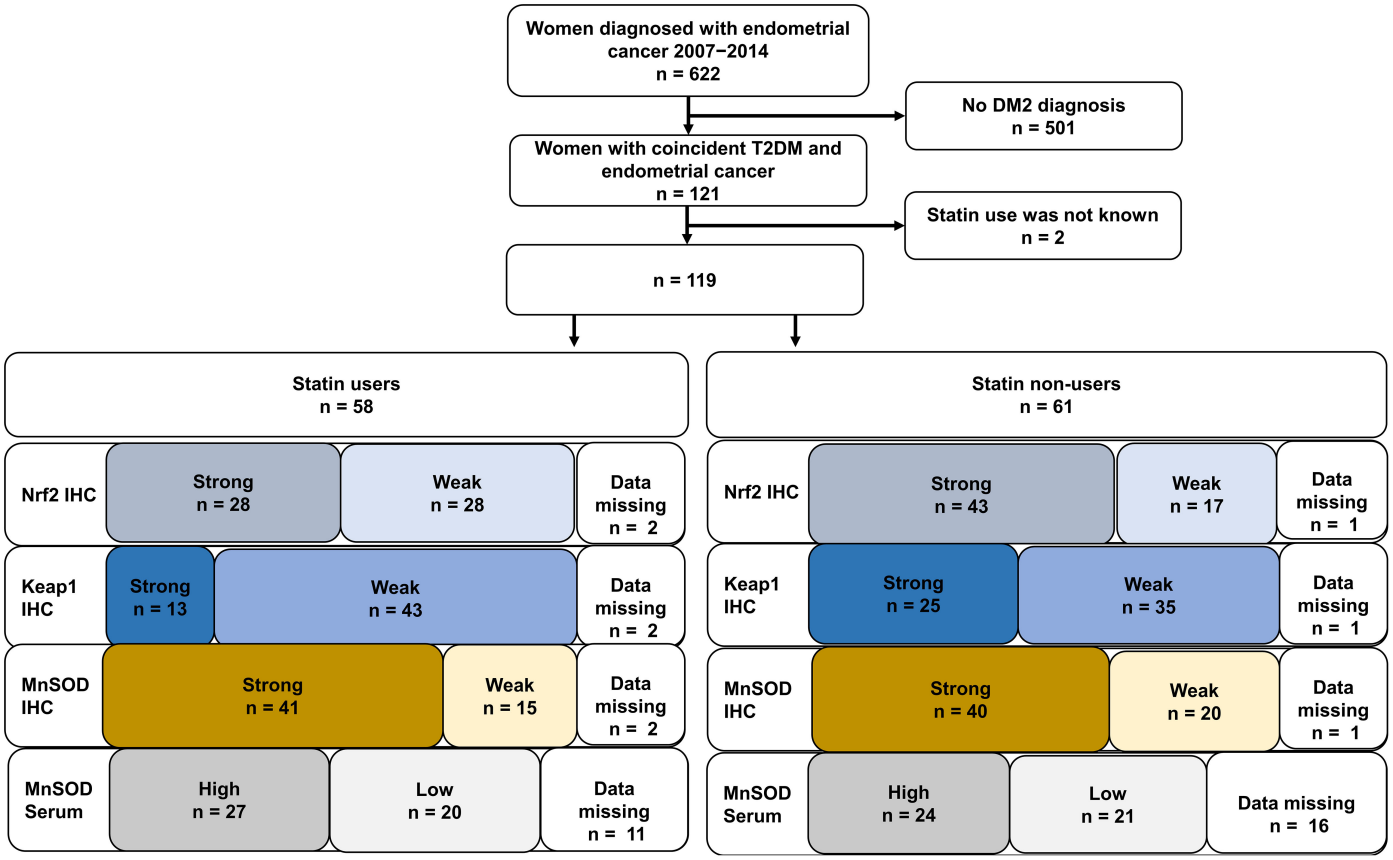
Flowchart with immunohistochemical expression and serum level distribution.

There were 121 women with T2DM diagnosed with endometrial cancer between 2007 and 2014 at Oulu University Hospital in Finland (**Figure 2**), and the median follow-up time was 63 months. Two patients were excluded because of a lack of information on statin use. At the time of endometrial cancer diagnosis, there were 58 statin users and 61 non-users. The most used statins were simvastatin (59%) and atorvastatin (19%).

Patient characteristics were similar in both weak and strong MnSOD tissue expression groups (**Table 1**), as well as in the groups defined by serum MnSOD levels. Keap1 expression was stronger (p = 0.03) and Nrf2 expression was weaker (p = 0.02) in the patients using statins.

**Table 1:** Patient characteristics according to the redox-state regulating proteins Nrf2 (Nuclear factor erythroid-related factor 2), Keap1 (Kelch-like ECH-associated protein 1) and MnSOD (Manganese superoxide dismutase).

	Nrf2 expression (IHC)			Keap1 expression (IHC)			MnSOD expression (IHC)			MnSOD level (serum)		
	Weak	Strong	p-value	Weak	Strong	p-value	Weak	Strong	p-value	Low	High	p-value
**Age at diagnosis (years)**			0.77			0.25			0.16			0.04
**Mean**	70.82	71.25		70.44	72.42		69.34	71.84		70.44	72.42	
**Range**	51–88	51–88		51–88	51–88		53–81	51–88		53–88	51–84	
**BMI (kg/m^2^)**			0.3			0.2			0.44			0.19
**Median**	34	35		33.50	37		34.50	34.00		35	33	
**Range**	19–51	22–65		19–55	22–65		23–55	19–65		23–55	22–48	
**Missing**	4	5		5	4		1	8		3	2	
**Parity**			0.53			0.89			0.77			0.77
**Median**	2.00	2.00		2.00	3.00		2.00	2.00		2.00	2.00	
**Range**	0–13	0–9		0–9	0–13		0–9	0–13		0–9	0–13	
**Missing**	0	3		0	3		1	2		0	1	
**Statin use**			0.01			0.03			0.44			0.69
**Yes**	28	28		43	13		15	41		20	27	
**No**	17	43		35	25		20	40		21	24	

IHC, Immunohistochemistry.

The tumor characteristics (**Table 2**) were also similar in the groups defined by MnSOD and Nrf2 expression, as well as MnSOD serum levels. Estrogen receptor positivity was more common in patients with weak Keap1 expression (p = 0.03). The majority of operated patients (97.96%) did not have any postoperative residual disease.

**Table 2:** Tumor characteristics according to the redox-state regulating proteins Nrf2 (Nuclear factor erythroid-related factor 2), Keap1 (Kelch-like ECH-associated protein 1) and MnSOD (Manganese superoxide dismutase).

	Nrf2 expression (IHC)			Keap1 expression (IHC)			MnSOD expression (IHC)			MnSOD level (serum)		
	Weak	Strong	p-value	Weak	Strong	p-value	Weak	Strong	p-value	Low	High	p-value
**Histology**			0.30			0.20			0.36			0.10
**Type 1**	31	55		55	31		24	24		33	33	
**Type 2**	14	16		23	7		11	19		8	18	
**Stage**			0.91			1.00			0.64			0.52
**Early**	33	47		56	24		23	57		28	38	
**Advanced**	12	18		21	9		10	20		12	12	
**Missing**	0	6		1	5		2	4		1	1	
**Deep Myometrial Invasion**			0.84			0.53			0.58			0.51
**Yes**	18	23		32	9		14	27		15	16	
**No**	26	36		45	17		18	44		23	33	
**Missing**	1	12		1	12		3	10		3	2	
**Lymphovascular invasion**			0.48			0.08			0.25			0.14
**Yes**	17	19		31	5		14	22		10	20	
**No**	26	39		46	19		18	47		27	27	
**Missing**	2	13		1	14		3	12		4	4	
**Estrogen receptor status**			0.18			0.03			0.44			0.66
**Positive**	37	59		60	36		30	66		35	42	
**Negative**	6	12		17	1		3	15		6	8	
**Missing**	2	0		1	1		2	0		0	1	

IHC, Immunohistochemistry.

Among the statin users, there were 43 (74%) cases with weak and 13 (22%) cases with strong Keap1 expression, 28 (48%) cases with weak and 28 (48%) cases with strong Nrf2 expression, and 15 (26%) cases with weak and 41 (70%) cases with strong MnSOD expression (**Figure 2**). In the serum samples, MnSOD levels were high in 27 (47%) patients and low in 20 (34%) patients.

In the statin non-users, there were 35 (57%) and 25 (41%) cases with weak and strong Keap1 expression, respectively. While there were 17 (28%) and 43 (70%) cases with weak and strong Nrf2 expression, respectively. Furthermore, there were 20 (33%) and 40 (66%) cases with weak and strong expression of MnSOD, respectively. MnSOD serum levels were high in 24 (39%) and low in 21 (34%) of statin non-users.

**Figure 3:**
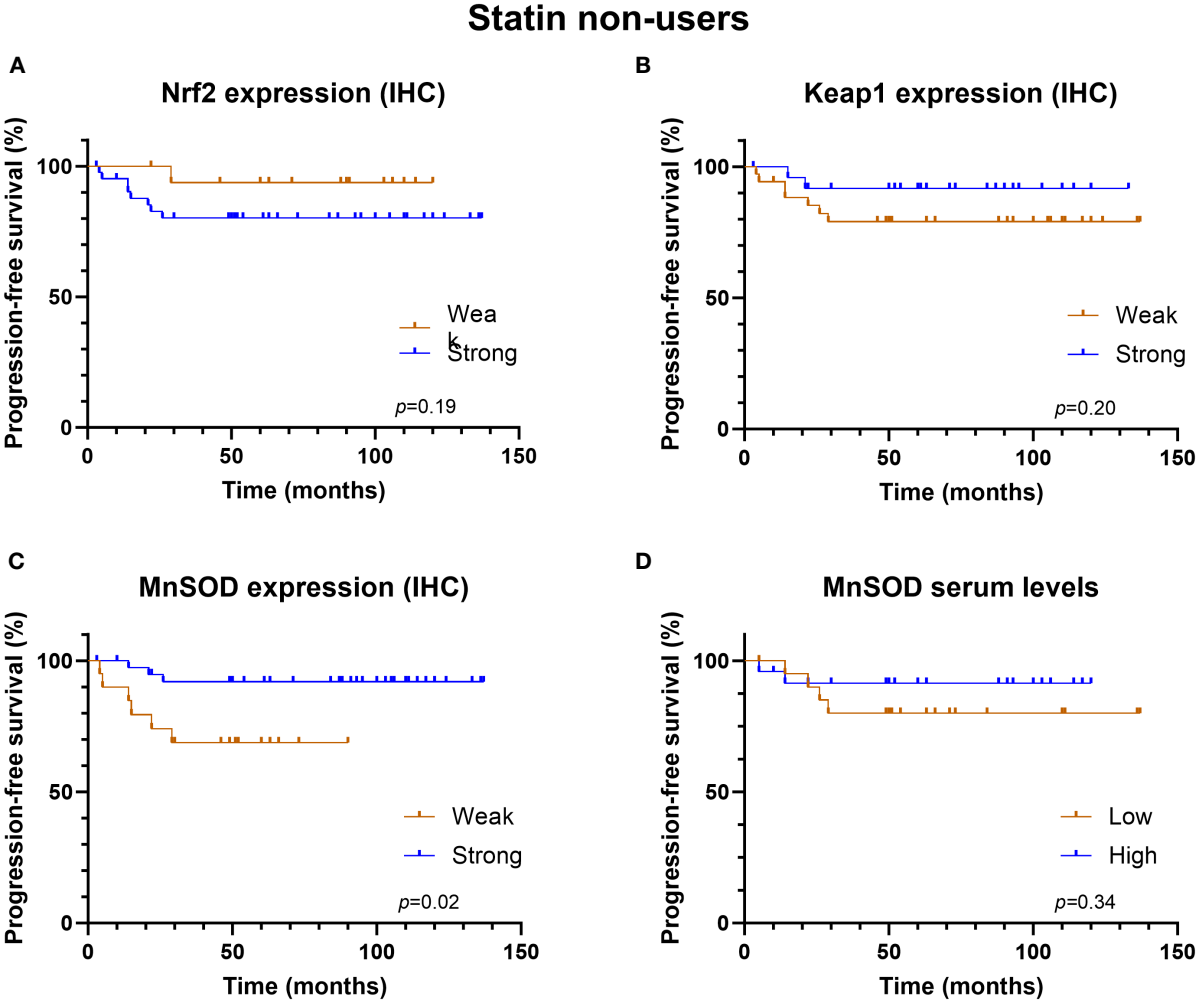
Kaplan-Meier progression-free survival graphs for statin non-users according to **(A)** Nrf2 expression, **(B)** Keap1 expression, **(C)** MnSOD expression and **(D)** MnSOD serum levels.

**Figure 4:**
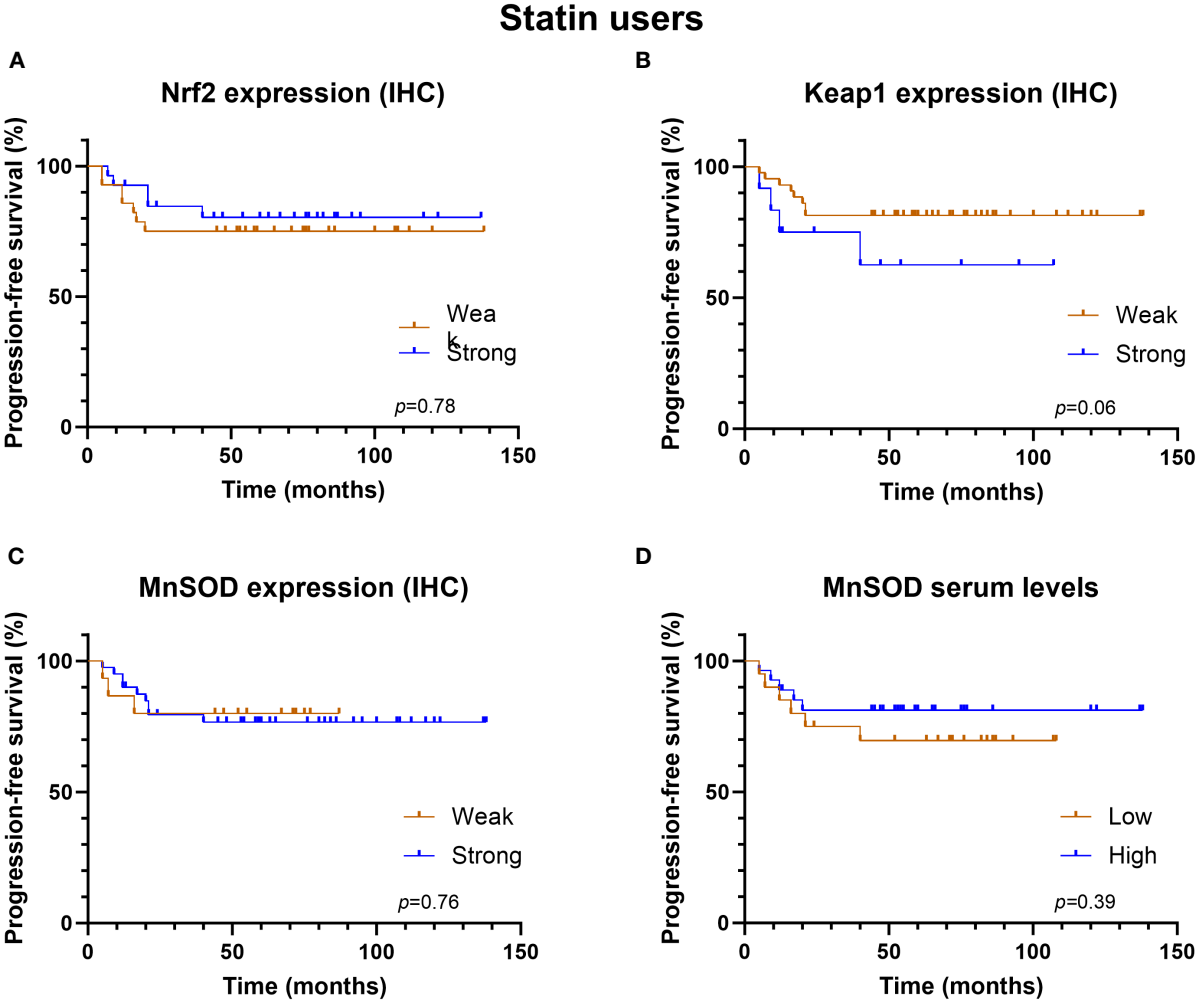
Kaplan-Meier progression-free survival graphs for statin users according to **(A)** Nrf2 expression, **(B)** Keap1 expression, **(C)** MnSOD expression and **(D)** MnSOD serum levels.

The univariate analysis revealed that the redox-state regulating proteins—Nrf2, Keap1, and serum MnSOD—were associated with neither PFS nor OS in the whole study cohort, not even in the subgroups defined by statin use. Interestingly, strong MnSOD expression was associated with better PFS in statin non-users (**Figure 3**, log rank p = 0.02) but not in statin users (**Figure 4**).

According to the Cox regression analysis, the redox-state regulating proteins were not associated with OS or PFS after adjusting for stage and statin use (**Table 3**). An advanced stage was the only clear indicator of poor prognosis regarding both PFS and OS.

**Table 3:** Progression-free survival and overall survival according to Cox regression analysis, considering stage, statin use, and the redox-state regulating proteins.

	Progression-free survival			Overall survival		
	Hazard ratio	95% Confidence interval	p-value	Hazard ratio	95% Confidence interval	p-value
**Stage (early vs advanced)**	21.44	6.16–74.70	<0.001	4.18	2.07–8.44	<0.001
**Statin (non-users vs users)**	1.05	0.41–2.71	0.91	1.03	0.51–2.09	0.94
**Nrf2 IHC (weak vs strong)**	0.87	0.34–2.23	0.77	0.94	0.47–1.91	0.87
**Stage (early vs advanced)**	21.12	6.10–73.60	<0.001	4.09	2.03–8.24	<0.001
**Statin (non-users vs users)**	1.09	0.44–2.75	0.85	1.09	0.54–2.91	0.81
**Keap1 IHC (weak vs strong)**	1.03	0.37–2.88	0.96	1.44	0.71–2.95	0.31
**Stage (early vs advanced)**	21.19	6.11–73.51	<0.001	4.14	2.07–8.28	<0.001
**Statin (non-users vs users)**	1.17	0.47–2.93	0.74	1.07	0.54–2.13	0.85
**MnSOD IHC (weak vs strong)**	0.54	0.21–1.34	0.18	0.71	0.33–1.52	0.38
**Stage (early vs advanced)**	28.66	6.45–127.40	<0.001	4.9	2.25–10.67	<0.001
**Statin (non-users vs users)**	1.92	0.67–5.56	0.23	1.29	0.59–2.82	0.53
**MnSOD serum level (low vs high)**	0.68	0.24–1.90	0.46	1	0.47–2.18	0.99

MnSOD, Manganese superoxide dismutase; Keap1, Kelch-like ECH-associated protein 1; Nrf2, Nuclear factor erythoid–related factor 2; IHC, Immunohistochemistry.

## 4 Discussion

In this study, we explored the role of cellular redox-state regulating proteins and statin use on endometrial cancer in women with coincident T2DM. Statin use seems to have effect on MnSOD expression in patients with T2DM and endometrial cancer since the statin non-user group with high MnSOD expression had better PFS compared to those with weak expression. This finding did not apply to statin users. However, MnSOD expression was not associated with prognosis when analyzing the whole study group.

Previous studies of oxidative stress markers and antioxidant enzymes have involved either patients with T2DM or patients with endometrial cancer. Our study was the first to focus on patients with T2DM and coincident endometrial cancer. According to earlier studies involving patients with endometrial cancer, strong Nrf2 and Keap1 expression is related to poorer prognosis (11). However, this finding was not reaffirmed in the current study and underlying T2DM diagnosis might be one of the explanations.

MnSOD can affect cancer prognosis in various ways. MnSOD is encoded by the nuclear SOD2 gene, which is regulated in various ways (e.g., by growth factors). SOD2 gene regulation in cancer cells depends on mutations, epigenetic processes, and the expression of repressive transcription processes (16). SOD2 Val-9Ala polymorphism, which results in more efficient importation of the MnSOD enzyme, seems to increase prostate and cervical cancer risk (17) while not affecting ovarian cancer risk or survival (18). MnSOD overexpression inhibits angiogenesis in both melanoma and endothelial cells of mice (19).

MnSOD expression has been proven to suppress tumors by controlling superoxide radical anions (20). Contrary to our findings, high MnSOD expression was observed in aggressive triple-negative breast cancer cells and was regarded as a poor prognostic marker by promoting an immunosuppressive tumor microenvironment (12). According to a TissueScan sDNA study, strong MnSOD expression was measured in 80% of tissue samples of breast cancer and none of the normal healthy breast tissues expressed strong MnSOD (12). Also, strong expression of MnSOD in patients with endometrioid ovarian carcinoma has been associated with poor PFS and OS (21). A mice study showed that MnSOD expression is weak in the early stages of cancer and is stronger in more advanced stages of skin cancer (13). This association between tumor aggressiveness and high MnSOD levels has also been shown in triple-negative breast cancer as well as colon and prostate cancer. High MnSOD levels are associated with OS in triple-negative breast cancer as well (22). MnSOD expression can suppress tumor initiation but, in later stages, promotes metastasis and invasion in breast cancer (23). In our study, with invasive cancer only, MnSOD was not associated with cancer stage. The variable results regarding the association with strong expression of MnSOD and endometrial cancer prognosis is one of the reasons the Human Protein Atlas database does not categorize MnSOD as a prognostic marker (24). Further studies on MnSOD level changes between early and advanced stages of endometrial cancer are required.

Although cholesterol synthesis itself is minimal in tumor cells, statin treatment downregulates the mevalonate-pathway product coenzyme Q which leads to severe oxidative stress and ROS production in tumor cells (25). On the other hand, statins may induce protective effects against oxidative stress by upregulating serum superoxide dismutase levels (26), and the results were similar after fluvastatin, atorvastatin and simvastatin treatment (27). Furthermore, simvastatin seems to partially restore renal levels of superoxide dismutase and reduce oxidative biomarker levels in animals with diabetes (28).

It is still debated whether statin users have a better cancer prognosis. A large meta-analysis, which included uterine cancer studies, showed that statin use was associated with a lower risk of all-cause mortality (29). Our own study of the same study population showed that there was no association between statin use and endometrial cancer prognosis in women with T2DM (30). There is also a difference in the type of statins patients use. A meta-analysis of patients with breast cancer showed that lipophilic statins lower both breast cancer-specific and all-cause mortality (31).

The strengths of this study were its long follow-up period and trustworthy data from a single institution. The data were comprehensive and included both patient characteristics (including body mass index, parity, and menopause age) and tumor characteristics (ER status, myometrial invasion, and histology type). Furthermore, we were able to study several factors that impacted cellular redox state regulation.

The main limitation of this study was its small cohort size, which limited the power of the outcome analysis. Lack of information about the cause of death and the severity of diabetes was also a shortcoming. We did have information on statin use at the time of the diagnosis of endometrial cancer, but the data on duration of statin use after the diagnosis as well as the dose of used statin were not available. Data concerning patient cardiovascular diseases as well as patient LDL levels would have been valuable. Due the small sample size, we did not adjust the data according to the type of statin used. Different kind of statins are known to differ in their absorption, bioavailability, plasma protein binding, excretion as well as their lipophilicity (32). It is debated whether hydrophilic or lipophilic statin is better for long PFS and OS. However, the vast majority of statins used in this study were lipophilic simvastatin and atorvastatin. It is also shown that statin use increases the risk for dysglycemia (32) and T2DM development dose dependently (33). Furthermore, long-term statin exposure associate with elevated T2DM risk (33). It seems that some statins (e.g. simvastatin and atorvastatin) are more diabetogenic than others (33). Multiple mechanisms seem to cause β-cell dysfunction and peripheral tissues’ insulin resistance in statin treatment (34). It is also suggested that statins’ interference with mitochondrial pathways might be the reason for adverse effects, e.g. development of diabetes (35). Our study cohort consisted solely from patients with T2DM but, unfortunately, we were not able to identify those statin users whose hypercholesterolemia was diagnosed before T2DM. Due to the timeframe of pathological diagnosis in 2007–2014, the modern 5th edition of WHO Classification of Tumors (15) was not used and the molecular classification of tumors to POLE-ultramutated, MMRd, NSMP or p53-mutant categories (36) was not obtained. Moreover, the classification of endometrial tumors to type 1 and type 2 according to histology and immunohistochemistry is well established in the field and the results are comparable to the earlier studies.

In conclusion, our study demonstrated that statin use effects on redox-state regulating proteins. Strong expression of MnSOD resulted in better PFS in the statin non-user group. There was no statistical difference in PFS between the groups with strong and weak expressions of Nrf2 and Keap1. These findings are preliminary and require further research.

## Data availability statement

The raw data supporting the conclusions of this article will be made available by the authors, without undue reservation.

## Ethics statement

The studies involving humans were approved by the Local Ethics Committee of Oulu University Hospital and the National Supervisory Authority for Welfare and Health. The studies were conducted in accordance with the local legislation and institutional requirements. The participants provided their written informed consent to participate in this study.

## Author contributions

EO: Writing – original draft, Formal analysis, Methodology, Software. AA: Conceptualization, Data curation, Methodology, Investigation, Writing – review & editing. UP: Conceptualization, Writing – review & editing. PK: Writing – review & editing. EU: Conceptualization, Data curation, Investigation, Methodology, Visualization, Writing – review & editing.
